# Biocontrol of *Aspergillus* and *Fusarium* Mycotoxins in Africa: Benefits and Limitations

**DOI:** 10.3390/toxins11020109

**Published:** 2019-02-13

**Authors:** Victor Kagot, Sheila Okoth, Marthe De Boevre, Sarah De Saeger

**Affiliations:** 1 MYTOX-SOUTH, Centre of Excellence in Mycotoxicology and Public health, Ghent University, 9000 Ghent, Belgium; victor.kagot@ugent.be (V.K.); sarah.desaeger@ugent.be (S.D.S.); 2 Centre for Biotechnology and Bioinformatics, University of Nairobi, Nairobi 00100, Kenya; dorisokoth@yahoo.com

**Keywords:** aflatoxins, fumonisins, biocontrol, *Aspergillus*, *Fusarium*, Africa

## Abstract

Fungal contamination and the consequent mycotoxin production is a hindrance to food and feed safety, international trade and human and animal health. In Africa, fungal contamination by *Fusarium* and *Aspergillus* is heightened by tropical climatic conditions that create a suitable environment for pre- and postharvest mycotoxin production. The biocontrol of *Fusarium* and its associated fusariotoxins has stagnated at laboratory and experimental levels with species of *Trichoderma*, *Bacillus* and atoxigenic *Fusarium* being tested as the most promising candidates. Hitherto, there is no impetus to upscale for field use owing to the inconsistent results of these agents. Non-aflatoxigenic strains of *Aspergillus* have been developed to create biocontrol formulations by outcompeting the aflatoxigenic strains, thus thwarting aflatoxins on the target produce by 70% to 90%. Questions have been raised on their ability to produce other mycotoxins like cyclopiazonic acid, to potentially exchange genetic material and to become aflatoxigenic with consequent deleterious effects on other organisms and environments. Other biocontrol approaches to mitigate aflatoxins include the use of lactic acid bacteria and yeast species which have demonstrated the ability to prevent the growth of *Aspergillus flavus* and consequent toxin production under laboratory conditions. Nevertheless, these strategies seem to be ineffective under field conditions. The efficacy of biological agents is normally dependent on environmental factors, formulations’ safety to non-target hosts and the ecological impact. Biocontrol agents can only be effectively evaluated after long-term use, causing a never-ending debate on the use of live organisms as a remedy to pests and diseases over the use of chemicals. Biocontrol should be used in conjunction with good agricultural practices coupled with good postharvest management to significantly reduce mycotoxins in the African continent.

## 1. Introduction

Mycotoxins are secondary metabolites produced by filamentous fungi as a strategy to secure an ecological advantage over other organisms which share their ecosystem and enhance their fitness against extreme environments. Under specific environmental conditions, a variety of fungi such as *Aspergillus*, *Penicillium*, *Fusarium* and *Claviceps* spp. colonize their host and produce mycotoxins [1]. Over 300 mycotoxins have been identified, with approximately 30 being recognized to have adverse health effects on vertebrates upon ingestion [1,2]. Some of these toxins trigger immune deficiency, lower production in livestock and are carcinogenic [3]. Unfortunately, once contaminated, feed or food containing mycotoxins are condemned because these toxins are resilient and stable against thermal, physical and chemical treatments during food processing. This poses a hurdle in free trade and food security since contaminated food and feed ends up as discarded waste [2,3].

*Fusarium* and *Aspergillus* are aerobic fungi with oligotrophic capabilities. They have a geographically global distribution, mostly found in terrestrial habitats ranging from soil, plants, other organisms and human-made substrates [4,5]. In the environment, they play a crucial role in decomposition and the cycling of nutrients, and have the potential for exploitation in bioremediation, biocontrol and bio-detoxification [6,7]. In industry, members of the *Aspergillus* genus have been utilized to produce organic acids, extracellular enzymes and beneficial secondary metabolites like lovastatin [8], while members of the *Fusarium* genus are being used in the production of mycoproteins, distributed under the brand name Quorn as a meat-substitute in foods [9]. In human, animal and plant health, these fungi can cause diseases either through invasive growth, which is common and fatal in immunosuppressed organisms, or through the consumption of food or feed contaminated with mycotoxins [10,11]. Mycotoxins cause enormous economic losses ranging from loss of life, decreased production in animals and increased costs of veterinary and human health care services. Under severe contaminations, total losses are experienced when the produce is declared unfit for consumption, rejected by the market and consequently destroyed [12].

In Africa, approximately 0.5 billion people are at risk of chronic exposure to aflatoxins through the consumption of maize and other foodstuffs prone to *Aspergillus* proliferation. Aflatoxin exposure surveys have confirmed the presence of aflatoxins in infant blood samples from Ghana, Kenya, Nigeria, Sudan, Benin, Togo, Egypt and Gambia [13]. Parts of the continent have reported widespread stunting in children, immune suppression and in some cases child neurological impairment traits linked to chronic dietary consumption of aflatoxins [13,14,15]. In Kenya, loss of life has been reported repeatedly from acute aflatoxicosis [13,16,17]. Currently, mitigation measures in place include regulations which impose maximum limits or guidance values of mycotoxins in food and feed to avert future untimely loss of life from the consumption of mycotoxin-contaminated food. Several African countries have put up country-specific regulations which set maximum limits for aflatoxins, including Kenya, Egypt, Mozambique, Tanzania and Nigeria (sum of aflatoxins B1, B2, G1 and G2, 10 µg/kg). South Africa, Tunisia and Zimbabwe targeted this level at 5 µg/kg [18]. Available mitigation technologies like the use of hermetic storage is too expensive for small-scale farmers and the developing governments [12].

*Fusarium* mycotoxins have not been given much attention in Africa, despite correlation studies suggesting links between *Fusarium* and fumonisin incidence and increasing human esophageal carcinomas in parts of South Africa and Kenya [18,19]. Unlike aflatoxins, which have caused documented loss of life in Africa, the actual repercussions of consumption of fumonisins and other *Fusarium* mycotoxins remain inferential. Occurrences of fumonisins in food crops and products have been reported across Africa, with countries like Botswana, Burkina Faso, Cameroon, Congo, Ivory Coast, Ghana, Kenya, Malawi, Mozambique, Nigeria, Benin, South Africa, Zambia and Zimbabwe recording samples with fumonisin levels larger than 1000 µg/kg [4]. Despite the known occurrence and contamination levels of fumonisins, many African countries do not have regulations aimed at controlling and monitoring fumonisins or other *Fusarium* mycotoxins. Consequently, control strategies have stagnated at laboratory and experimental levels with no impetus to upscale for field use [4,19].

The mycotoxin problem in Africa only comes to the limelight when there is an outbreak and lives are lost. During that period, regulation is enforced and contaminated food and feed is impounded and destroyed. However, shortly after the regulation becomes unenforceable owing to weak non-sustainable institutions. In this part of the world, the population is at a serious risk of chronic exposure to mycotoxins since regulation is seldom enforced. Moreover, the poorest might prefer to consume cheap, physically damaged and visibly discolored maize which is potentially contaminated with mycotoxins. The anxiety of future repercussions is not taken into account when faced with the prospect of starvation [20].

The burden of chronic exposure in humans to mycotoxins in Africa is a matter of scientific discourse since there is a lack of data to ascertain the level of harm that the toxins actually cause. Emerging control strategies have not been adopted by farmers owing to their inaccessibility and the prohibiting cost. Besides, no single mitigation measure has proven to be robust enough for wide-scale adoption in Africa—each approach has its own merits and demerits. These interventions can be divided into pre- and postharvest, and successful control strategies should encompass approaches which control toxins both preharvest/farm and postharvest/storage [21]. An upcoming preharvest management strategy is breeding, which employs both conventional and transgenic technologies to identify and enhance plants with resistance to mycotoxin contamination and fungal ear rot. So far, using such efforts, the International Crops Research Institute for the Semi-Arid Tropics (ICRISAT) has reported a peanut variety resistant to aflatoxin contamination [22]. In similar studies by Okoth et al. (2017), conducted in Kenya and South Africa, maize inbred lines CML247, CML444, CML495, LaPosta, CML 390 and CB222 demonstrated resistance to *Aspergillus* ear rot and aflatoxin contamination. Some of these lines like CB222, CML444 and CML390 were resistant not only to aflatoxins, but also to fumonisins and *Fusarium* ear rot [23].

Governments across Africa have intensified campaigns on good agricultural practices as a strategy to reduce the toxin load in food. Farmers are being urged to select healthy seeds, practice crop rotation, plant in good time (just at the onset of rain) and maintain optimal seed density. These are factors which generally reduce the fungal load in the soil and consequent toxin contamination. [24]. The use of biocontrol agents as a strategy against the toxins has emerged as a strong preharvest method as it has consistently reduced aflatoxin contamination by 70% to 90% both in laboratory and field trials [25].

Postharvest methods include proper drying and the use of hermetic storage devices. These have been shown to possess the capability to arrest the mycotoxin increase in the stored grains by depriving the toxigenic fungus of oxygen, hence preventing further development and consequent toxin production [21]. Sorting options have been suggested and tested using near-infrared hyperspectral imaging devices to detect aflatoxin-contaminated maize and peanuts [26]. Smallholder farmers are encouraged to practice hand sorting where physically infected grains are removed, which can significantly reduce mycotoxin concentration in the sorted grain [27]. The use of binders is a promising strategy since it is being practiced in the livestock and poultry industry. Binders reduce the bioavailability of mycotoxins in the gastrointestinal tract by adhering to the toxins, rendering them not absorbable [28]. The use of binders in humans has been tried in Africa. In Ghana, NovaSil clay was effective in decreasing exposure to aflatoxin [29]. In Kenya, the Centre for Disease Control (CDC) conducted binder trials using human subjects with promising results in 2015 [13]. The use of binders has had skeptics questioning effects of binders on the adsorption of other nutrients and minerals essential for normal body functioning.

Stakeholders in Africa have only focused on aflatoxins with little or no attention given to other mycotoxins, yet analysis pinpoints the existence of fumonisins among other toxins. Farmers, despite being aware of the threats posed by mycotoxins, are slow to adopt any mycotoxin mitigation technology which involves them spending more money since the contamination process is invisible, and the produce does not fetch an increased premium for being toxin-free. Often of importance to the farmer is controlling insect damage and maintaining high grain quality parameters which might attract a high price in the market [21]. Research has revealed the negative impacts of fumonisins and aflatoxins on human and animal health, food security and trade across Africa [13,19]. The fear exists that millions are consuming doses of these mycotoxins in their daily food intake.

Several technologies are being tested and promoted for mycotoxin management. The use of biocontrol agents as a strategy against mycotoxins is rapidly being adopted across African nations. This opinion paper attempts to analyze the types of biocontrol on *Aspergillus* and *Fusarium* mycotoxins present on the African continent, the extent of adoption and possible challenges facing this technology in Africa.

## 2. Biocontrol of *Aspergillus* Mycotoxins in Africa

The *Aspergillus* genus consists of a diverse assemblage of microorganisms that produce a wide range of mycotoxins. *A. versicolor* and *A. nidulans* produce sterigmatocystin; *A. ochraceus* produces ochratoxin, citrinin, viomellein and penicillic acid; and *A. clavatus* produces patulin. Other metabolites synthesized by the fungus include aflatrem, aflavinine, kojic acid, flavocol, aspergillic acid, aspertoxin, cyclopiazonic acid, paspallinine and aflatoxins [30]. On the other hand, *A. terreus* produces lovastatin, an anti-cholesterol agent, and *A. fumigatus* produces verruculogen and helvolic acid, which exhibit anti-fungal activities. Aflatoxins are the most potent natural carcinogens and up to 20 members of the *Aspergillus* genus—assigned to three sections, *Flavi*, *Nidulantes* and *Ochraceorosei*—have been reported to be toxigenic. *A. flavus* is the most virulent species in terms of toxin production [13,31,32,33,34]. The fungus exhibits a high genetic diversity within its populations, mostly as a result of sexual reproduction characterized by the formation of ascospore-bearing ascocarps inside sclerotia. Genetic recombination is possible between strains of opposite mating types since the fungus is heterothallic [35].

*A. flavus* comprises an assemblage of phylogenetically-related aflatoxin and non-aflatoxin producing strains with the toxin production varying dependent on the isolate. The fungus is divided into S and L morphotypes. The S morphotypes produce, on average, much higher aflatoxins than the L morphotypes. Each morphotype is further divided into vegetative compatibility groups (VCGs) determined by a series of *het* loci. Individuals belonging to compatible VCGs have successful hyphal fusion and transfer of genetic material, while the transfer of genetic material amongst individuals of incompatible groups is not possible. In this regard some isolates produce no aflatoxins at all, and are termed atoxigenic. The aflatoxin-producing ability tends to be similar among members of the same VCG [13,36]. Efforts to control aflatoxin contamination have involved the use of non-aflatoxigenic strains of *Aspergillus* as biocontrol agents to outcompete aflatoxigenic strains. In 1990, in the Arizona cotton fields, Cotty [37] demonstrated that wounded cotton bolls recorded lower levels of aflatoxin in the cotton seeds compared to cotton seeds of wounded bolls inoculated with aflatoxigenic strains alone while simultaneously inoculated with aflatoxigenic and non-aflatoxigenic *A. flavus* strains. In addition, aflatoxin levels were further reduced when non-aflatoxigenic strains were inoculated one day before inoculating aflatoxigenic strains. Also, the levels of aflatoxins did not reduce when aflatoxigenic strains were inoculated before their non-aflatoxigenic counterparts [37]. This phenomenon has been described as competitive exclusion, where the non-aflatoxigenic strains effectively compete for space and nutrients, thus excluding their aflatoxigenic counterparts. 

Large-scale studies over the years have led to the development of biocontrol agents for commercial application based on the ability of non-aflatoxigenic strains to reduce aflatoxin contamination in cotton seeds, peanuts and corn. Such products include aflaguard^®^ and AF36^®^ registered by the USA Environmental Protection Agency, and Aflasafe^®^ registered in Nigeria and Kenya [11]. These formulations are based on the fact that non-aflatoxigenic strains outcompete aflatoxigenic strains, leading to their incapability to synthesize aflatoxins. Another factor is that aflatoxigenic and non-aflatoxigenic strains are selected based on their vegetative incompatibility, which limits sexual reproduction and exchange of genetic material through the parasexual cycle, ensuring the stability of the biocontrol formulation [38].

In both Kenya and Nigeria (where Aflasafe^®^ is commercialized), specific requirements had to be met before registration. In Nigeria, the National Agency for Food and Drug Administration and Control (NAFDAC) required that poultry feed on Aflasafe^®^-treated sorghum be investigated to assess the products’ safety. In Kenya, the Pest Control Products Board (PCPB) required toxicological and eco-toxicological studies to be conducted. In Senegal, Ghana, Burkina Faso, Tanzania and Zambia, the development of the formulations is at an advanced stage and are due for registration. In Mozambique and Malawi, the formulations are under testing in farmers’ fields, and in Ethiopia and Uganda, the formulations are at strain development. Other African countries like Mali and the D.R. Congo are also expressing their interest [39].

Lactic acid bacteria (*Lactobacillus* (*L.*) *casei* and *L. reuteri*) have demonstrated the ability to bind with aflatoxins in aqueous solutions. Hathout et al. (2011) observed that aflatoxin-induced stress in rats, leading to debilitating health and deteriorating liver functions, was restored to health by treatment with lactic acid bacteria. In other in vitro tests, *L. amylovorus* and *L. rhamnosus* demonstrated the ability to bind up to 60% AFB1, showing their potential to bind and lock-up selected dietary contaminants [40,41]. In similar tests *L. rhamnosus* reduced the uptake of AFB1 by 74% in chicken intestinal tissue, suggesting their potential for exploitation as toxin binders. Incorporating lactic acid bacteria in diets offers a feasible approach to reduce chronic exposure to aflatoxins [42]. In other laboratory assays, *Pseudomonas* spp. and *Bacillus* strains demonstrated the ability to inhibit growth of *Aspergillus* and consequent toxin production. Some of these soil bacteria were found to produce aflatoxin inhibitors [43]. However, these bacteria were not effective against aflatoxin production and fungal growth in field conditions owing to the difficulty in handling pure bacterial cultures under field conditions. In other assays, saprophytic yeasts like *Pichia anomala* and *Candida krusei* have demonstrated efficacy against *A. flavus* and aflatoxin production in the laboratory. The yeasts also possess aflatoxin binding capabilities similar to the lactic acid bacteria; nevertheless, they have reported little success in the field [43,44].

## 3. Biocontrol of *Fusarium* Mycotoxins in Africa

*Fusarium* is a genus of filamentous fungi with perfect state members belonging to the genera *Gibberella*, *Calonectria*, *Nectria* and *Microneciriella*. The fungus is ubiquitous, and a notorious plant-pathogen to most food crops causing annual losses of millions of US dollars. In addition to causing an array of plant diseases, some members of the *Fusarium* genus produce deleterious secondary metabolites such as fumonisins, zearalenone and trichothecenes, known to cause devastating health complications in humans and animals [4,45].

Several biological control agents have been tested as suitable candidates against fumonisin-producing *F. verticilloides*. The strategy is to have a biological formulation which can outcompete the toxin-producing strains and end up with a toxin-free product. Atoxigenic *F. verticilloides* showed great promise in excluding their toxigenic counterparts; however, since they are plant pathogens, they resulted in a higher disease incidence [19]. The use of endophytic bacteria *Bacillus subtilis* demonstrated potential in precluding fungal hyphae and consequent toxin production. The bacteria reduced the amount of fumonisins produced by up to 50% since the bacteria is an ecological homologue to the fungus and it inhibits fungal growth through the competitive exclusion principle [46]. When *Bacillus amyloliquefaciens* and *Microbacterium oleovorans* were applied as seed coatings at a concentration of 10^7^ colony-forming units per millimeter, both had the ability to reduce fumonisin B1 and B2 in maize grains [47]. *Trichoderma* strains have shown good antibiosis and parasitism ability for exploitation as mycoparasites against toxigenic *Fusarium* isolates. A dual-culture bioassay on potato dextrose agar medium showed that *Trichoderma* outcompeted *Fusarium* by forming coils around the *Fusarium* hyphae, penetrating it and thus preventing the growth [19,48].

In vitro tests on wheat and maize grains showed that inoculation with *Microsphaerosis* species reduces *Gibberella zeae* ascospore production by 73% [49]. In other tests, inoculation of wheat ears with *Phoma betae* under glass house conditions reduced the severity of *Fusarium* head blight by up to 60%. Under similar conditions, *Pythium ultimum* induced a prolonged latency in *F. nivale*, delaying the disease onset [49]. *Bacillus* spp. have also been tested under glass house conditions and the *Bacillus* strain AS 43.4 was found to decrease the concentration of deoxynivalenol (DON) in grain by 89–97% [49]. In other laboratory tests rhizobacteria including *Azotobacter*, *Bacillus*, *Pseudomonas* and *Arthrobacter* reduced fumonisin B1 production and showed an ability to thwart *F. verticolloides* [50]. These tests demonstrate a potential for the biocontrol of *Fusarium* and consequently its mycotoxins. However, in field conditions these antagonistic strains give inconsistent results. The need for more research on their potential upscale and field use in Africa is imperative.

## 4. Potential Limitations and Benefits

Benefits of biocontrol formulations as a remedy against mycotoxins and especially aflatoxins in Africa have been documented as this strategy has reduced aflatoxin contamination by 70% to 90% both in laboratory and field trials [25]. However, this approach has attracted scrutiny from stakeholders in industry, academia and local governments who question its safety, sustainability, effects on other mycotoxins and its impact on the ecosystem [39]. A critique of this approach would be the fact that these biocontrol agents are being selected based on their inability to produce aflatoxins. However, both non-aflatoxigenic and aflatoxigenic *Aspergillus* strains have the ability to produce other mycotoxins or metabolites detrimental to humans and animals upon consumption. An example is cyclopiazonic acid (α-CPA), which is an enzyme (ATP-ase) inhibitor with the potential to affect normal muscle contraction and relaxation produced by the non-aflatoxigenic *Aspergillus* AF36, formally used in biocontrol formulations to mitigate aflatoxins in the USA. Currently, other non-aflatoxigenic strains which cannot synthesize α-CPA are being utilized as biocontrol agents in replacement of AF36 [51]. However, it should be taken into consideration that other CPA derivatives such as indole derivatives, speradines, aspergillines and cyclopiamides do exist. Hence, a more thorough (toxicological) assessment of these fungal metabolites is needed [52]. Moreover, *A. flavus* strains can synthesize an array of metabolites with unknown toxicological effects such as kojic acid, aflavinine, aspertoxin, aflatrem, paspalinine, leporin C and sterigmaticystin [30]. In each country where this technology is in use, country-specific regulations must be met prior to registration and adaptation. Fears of potential harm are reduced by risk assessment tests focusing on the biocontrol formulations as allergens to the skin and eye as well as effects on inhalation [39].

In addition, the use of biocontrol agents (e.g., Trichoderma) can affect biosynthetic fungal metabolic pathways leading to the conversion of mycotoxins into modified derivatives. The occurrence of these modified forms eventually leads to an underestimation of the overall mycotoxin content in a specific crop.

Recent studies have reported the existence of the sexual state of *Aspergillus* with sexual reproduction being observed in individuals belonging to different VCGs. Further, crossing sexually compatible strains in the laboratory has shown that the ability to produce aflatoxins can be passed from parent to progeny, suggesting the possibility that a non-aflatoxigenic strain can become toxigenic through sexual reproduction [53]. Nonetheless, if this occurs it is also possible that the atoxigenic strains can transfer atoxigenicity to their toxigenic counterparts and yield progenies without the ability to produce aflatoxins [39]. Questions on effects of introducing live microbes to the environment and the potential harm that the introduced inoculum could pose to other microbes and not target organisms have been raised. In this case, these biocontrol formulations should contain microorganisms native to the area of application, which potentially pose no harm to unintended individuals [39]. The influence of these biocontrol formulations on other mycotoxins—particularly fumonisins—is of concern [54]. In this regard, the use of atoxigenic *Aspergillus* strains against their toxigenic counterparts is primarily designed to tackle aflatoxins, with no reduction expected on other mycotoxins. Tests carried out by the developer in Nigeria between 2009 and 2012 demonstrated that the application of Aflasafe^®^ did not significantly affect the occurrence of fumonisins in treated and control fields [39]. More tests on safety and sustainability should, however, be elaborated to eradicate any doubt on potential deleterious effects on human and plant health and the environment.

## 5. Conclusions

Although the use of biocontrol formulations as a strategy against mycotoxins has raised concern, this approach has shown an ability to reduce aflatoxin contamination in grains. As with any new biocontrol technology, its actual effects can only be evaluated after long-term use. In the meantime, all stakeholders should be equipped with adequate information on the handling, application and potential risks posed by these biological formulations. Moreover, biocontrol alone will not be sufficient but should be used in conjunction with good agricultural practices coupled with good postharvest management like sorting and proper storage, preferably in hermetic devices to significantly reduce mycotoxins in the African continent. Research and development on these formulations should not cease so as to ensure the sustainability and safety of these formulations.

## References

[1] Alassane-Kpembi I., Schatzmayr G., Taranu I., Marin D., Puel O., Oswald I.P. (2017). Mycotoxins co-contamination: Methodological aspects and biological relevance of combined toxicity studies. Crit. Rev. Food Sci. Nutr..

[2] Alshannaq A., Yu J.H. (2017). Occurrence, toxicity, and analysis of major mycotoxins in food. Int. J. Environ. Res. Public Health.

[3] Kowalska A., Walkiewicz K., Koziel P., Muc-Wierzgon M. (2017). Aflatoxins: Characteristics and impact on human health. Postep. Hig. Med. Dosw..

[4] Chilaka C.A., De Boevre M., Atanda O.O., De Saeger S. (2017). The status of *Fusarium* mycotoxins in sub-saharan africa: A review of emerging trends and post-harvest mitigation strategies towards food control. Toxins.

[5] Paulussen C., Hallsworth J.E., Alvarez-Perez S., Nierman W.C., Hamill P.G., Blain D., Rediers H., Lievens B. (2017). Ecology of aspergillosis: Insights into the pathogenic potency of *Aspergillus fumigatus* and some other *Aspergillus* species. Microb. Biotechnol..

[6] Chhaya U., Gupte A. (2013). Possible role of laccase from *Fusarium incarnatum* uc-14 in bioremediation of bisphenol a using reverse micelles system. J. Hazard. Mater..

[7] Srivastava S., Thakur I.S. (2006). Biosorption potency of *Aspergillus niger* for removal of chromium (vi). Curr. Microbiol..

[8] Bennett J.W. (2010). An overview of the Genus *Aspergillus*. Aspergillus: Molecular Biology and Genomics.

[9] Ahangi Z., Shojaosadati S.A., Nikoopour H. (2008). Study of mycoprotein production using *Fusarium oxysporum* ptcc 5115 and reduction of its rna content. Pak. J. Nutr..

[10] Dweba C., Figlan S., Shimelis H., Motaung T., Sydenham S., Mwadzingeni L., Tsilo T. (2017). *Fusarium* head blight of wheat: Pathogenesis and control strategies. Crop Prot..

[11] Amaike S., Keller N.P. (2011). Aspergillus Flavus. Annu. Rev. Phytopathol..

[12] Okello D.K., Kaaya A.N., Bisikwa J., Were M., Oloka H.K. (2010). Management of aflatoxins in groundnuts. A Manual for Processors and Traders in Uganda.

[13] Okoth S. (2016). Improving the Evidence Base on Aflatoxin Contamination and Exposure in Africa.

[14] Bhat Ramesh V., Vasanthi S. (2003). Mycotoxin Food Safety Risk in Developing Countries.

[15] Mutegi C., Ngugi H., Hendriks S., Jones R. (2009). Prevalence and factors associated with aflatoxin contamination of peanuts from Western Kenya. Int. J. Food Microbiol..

[16] Probst C., Njapau H., Cotty P.J. (2007). Outbreak of an acute aflatoxicosis in Kenya in 2004: Identification of the causal agent. Appl. Environ. Microbiol..

[17] Wagacha J.M., Mutegi C., Karanja L., Kimani J., Christie M.E. (2013). Fungal species isolated from peanuts in major Kenyan markets: Emphasis on *Aspergillus* section *flavi*. Crop Prot..

[18] AC04318739 (2004). A Worldwide Regulations for Mycotoxins in Food and Feed in 2003.

[19] Wagacha J., Muthomi J. (2008). Mycotoxin problem in Africa: Current status, implications to food safety and health and possible management strategies. Int. J. Food Microbiol..

[20] Bandyopadhyay R., Kumar M., Leslie J.F. (2007). Relative severity of aflatoxin contamination of cereal crops in West Africa. Food Addit. Contam..

[21] Walker S., Jaime R., Kagot V., Probst C. (2018). Comparative effects of hermetic and traditional storage devices on maize grain: Mycotoxin development, insect infestation and grain quality. J. Stored Prod. Res..

[22] Nigam S., Waliyar F., Aruna R., Reddy S., Kumar P.L., Craufurd P., Diallo A., Ntare B., Upadhyaya H. (2009). Breeding peanut for resistance to aflatoxin contamination at ICRISAT. Peanut Sci..

[23] Okoth S., Rose L.J., Ouko A., Beukes I., Sila H., Mouton M., Flett B.C., Makumbi D., Viljoen A. (2017). Field evaluation of resistance to aflatoxin accumulation in maize inbred lines in Kenya and South Africa. J. Crop Improv..

[24] Waliyar F., Reddy S., Kumar P. (2005). Estimation of Aspergillus Flavus Infection and Aflatoxin Contamination in Seeds: Laboratory Manual.

[25] Marechera G., Ndwiga J. (2015). Estimation of the potential adoption of aflasafe among smallholder maize farmers in lower Eastern Kenya. Afr. J. Agric. Res. Econ..

[26] Wang W., Ni X., Lawrence K.C., Yoon S.-C., Heitschmidt G.W., Feldner P. (2015). Feasibility of detecting aflatoxin b 1 in single maize kernels using hyperspectral imaging. J. Food Eng..

[27] Matumba L., Van Poucke C., Njumbe Ediage E., Jacobs B., De Saeger S. (2015). Effectiveness of hand sorting, flotation/washing, dehulling and combinations thereof on the decontamination of mycotoxin-contaminated white maize. Food Addit. Contam. Part A.

[28] Huwig A., Freimund S., Käppeli O., Dutler H. (2001). Mycotoxin detoxication of animal feed by different adsorbents. Toxicol. Lett..

[29] Phillips T., Afriyie-Gyawu E., Williams J., Huebner H., Ankrah N.-A., Ofori-Adjei D., Jolly P., Johnson N., Taylor J., Marroquin-Cardona A. (2008). Reducing human exposure to aflatoxin through the use of clay: A review. Food Addit. Contam..

[30] Okoth S., De Boevre M., Vidal A., Diana Di Mavungu J., Landschoot S., Kyallo M., Njuguna J., Harvey J., De Saeger S. (2018). Genetic and toxigenic variability within *Aspergillus flavus* population isolated from maize in two diverse environments in kenya. Front. Microbiol..

[31] Bennett J. (2009). *Aspergillus*: A primer for the novice. Med. Mycol..

[32] Li X.-J., Zhang Q., Zhang A.-L., Gao J.-M. (2012). Metabolites from *Aspergillus fumigatus*, an endophytic fungus associated with melia azedarach, and their antifungal, antifeedant, and toxic activities. J. Agric. Food Chem..

[33] Scudamore K.A. (1994). *Aspergillus* toxins in food and animal feedingstuffs. The Genus Aspergillus.

[34] Klich M.A. (2007). *Aspergillus flavus*: The major producer of aflatoxin. Mol. Plant Pathol..

[35] Horn B.W., Gell R.M., Singh R., Sorensen R.B., Carbone I. (2016). Sexual reproduction in *Aspergillus flavus* sclerotia: Acquisition of novel alleles from soil populations and uniparental mitochondrial inheritance. PLoS ONE.

[36] Horn B.W., Moore G.G., Carbone I. (2009). Sexual reproduction in *Aspergillus flavus*. Mycologia.

[37] Cotty P. (1990). Effect of atoxigenic strains of *Aspergillus flavus* on aflatoxin contamination of developing cottonseed. Plant Dis..

[38] Ehrlich K.C. (2014). Non-aflatoxigenic *Aspergillus flavus* to prevent aflatoxin contamination in crops: Advantages and limitations. Front. Microbiol..

[39] Bandyopadhyay R., Ortega-Beltran A., Akande A., Mutegi C., Atehnkeng J., Kaptoge L., Senghor A., Adhikari B., Cotty P. (2016). Biological control of aflatoxins in Africa: Current status and potential challenges in the face of climate change. World Mycotoxin J..

[40] Hathout A.S., Mohamed S.R., El-Nekeety A.A., Hassan N.S., Aly S.E., Abdel-Wahhab M.A. (2011). Ability of *Lactobacillus casei* and *Lactobacillus reuteri* to protect against oxidative stress in rats fed aflatoxins-contaminated diet. Toxicon.

[41] Peltonen K., El-Nezami H., Haskard C., Ahokas J., Salminen S. (2001). Aflatoxin b1 binding by dairy strains of lactic acid bacteria and bifidobacteria. J. Dairy Sci..

[42] El-Nezami H., Mykkänen H., Kankaanpää P., Salminen S., Ahokas J. (2000). Ability of lactobacillus and propionibacterium strains to remove aflatoxin b1 from the chicken duodenum. J. Food Prot..

[43] Jalili M. (2016). A review on aflatoxins reduction in food. Iranian J. Health Saf. Environ..

[44] Yin Y.-N., Yan L.-Y., Jiang J.-H., Ma Z.-H. (2008). Biological control of aflatoxin contamination of crops. J. Zhejiang Univ. Sci..

[45] Booth C. (1971). The Genus Fusarium.

[46] Bacon C.W., Yates I.E., Hinton D.M., Meredith F.J. (2001). Biological control of fusarium moniliforme in maize. Environ. Health Perspect..

[47] Pereira P., Nesci A., Etcheverry M. (2007). Effects of biocontrol agents on *Fusarium verticillioides* count and fumonisin content in the maize agroecosystem: Impact on rhizospheric bacterial and fungal groups. Biol. Control..

[48] Błaszczyk L., Basińska-barczak A., Ćwiek-kupczyńska H., Gromadzka K., Popiel D., Stępień Ł. (2017). Suppressive effect of *Trichoderma* spp. On toxigenic *Fusarium* species. Polish J. Microbiol..

[49] Pirgozliev S.R., Edwards S.G., Hare M.C., Jenkinson P. (2003). Strategies for the control of *Fusarium* head blight in cereals. Eur. J. Plant Pathol..

[50] Cavaglieri L., Orlando J., Rodriguez M., Chulze S., Etcheverry M. (2005). Biocontrol of *Bacillus subtilis* against *Fusarium verticillioides* in vitro and at the maize root level. Res. Microbiol..

[51] Abbas H., Zablotowicz R., Horn B., Phillips N., Johnson B., Jin X., Abel C. (2011). Comparison of major biocontrol strains of non-aflatoxigenic *Aspergillus flavus* for the reduction of aflatoxins and cyclopiazonic acid in maize. Food Addit. Contam..

[52] Uka V., Moore G.G., Arroyo-Manzanares N., Nebija D., De Saeger S., Diana Di Mavungu J. (2017). Unravelling the diversity of the cyclopiazonic acid family of mycotoxins in *Aspergillus flavus* by uhplc triple-tof hrms. Toxins.

[53] Moore G.G., Elliott J.L., Singh R., Horn B.W., Dorner J.W., Stone E.A., Chulze S.N., Barros G.G., Naik M.K., Wright G.C. (2013). Sexuality generates diversity in the aflatoxin gene cluster: Evidence on a global scale. PLoS Pathog..

[54] Alberts J., Lilly M., Rheeder J., Burger H., Shephard G., Gelderblom W. (2017). Technological and community-based methods to reduce mycotoxin exposure. Food Control.

